# Supplementary material for: Functional outcomes after the treatment of hip fracture
Source: PLoS One. 2020 Jul 30;15(7):e0236652. doi: 10.1371/journal.pone.0236652 (PMC7392284; doi:10.1371/journal.pone.0236652)
Supplement: S1 File — (PDF) [file pone.0236652.s001.pdf]

	age	sex M1W2	type N1T2	L/R R1L2	treatment BHA1ORIF2conseva3	pre-op. days	hospital days	pre-op. FAC	admission FAC	6M later FAC unknown-dead-1	start walking from opration	pneumo Y1N2	urinary infe Y1N2	DVT/PE Y1N2	drug Y1N2	DM Y1N2
	81.71					7.77202073	20.76576577									
1	91	2	2	2	2	1	11	4	0	0	0	2	2	2	2	2
2	79	1	1	2	2	1	12	−	0	−	−	2	2	2	2	2
3	94	2	1	1	3	−	−	4	0	0	−	2	2	2	2	2
4	54	2	1	1	2	3	17	5	0	−	10	2	2	2	2	2
5	76	2	1	2	1	5	23	5	2	4	9	2	2	2	2	2
6	89	2	1	1	3	−	−	1	0	−	0	2	2	2	2	2
7	64	2	1	1	3	−	−	4	0	3	0	2	2	2	2	2
8	86	2	1	1	1	−	−	3	0	3	6	2	2	2	2	2
9	80	2	1	1	1	7	20	5	3	4	6	2	2	2	2	2
10	74	2	1	1	1	5	24	5	4	4	3	2	2	2	2	2
11	84	1	1	1	2	7	14	4	3	4	1	2	2	2	2	2
12	68	1	1	1	1	17	33	5	3	5	2	2	2	2	2	1
13	75	2	1	1	1	19	30	3	3	3	7	1	1	2	1	2
14	66	2	1	2	3	−	−	5	0	5	0	2	2	2	2	2
15	52	2	1	1	1	4	34	5	3	4	3	2	2	2	2	2
16	80	2	1	1	1	9	24	4	3	4	9	2	2	2	2	2
17	75	1	1	2	1	10	40	5	0	4	0	1	2	2	2	2
18	81	2	1	1	1	5	17	5	4	5		2	2	2	2	2
19	86	2	1	2	1	9	28	4	3	3	2	2	2	2	2	2
20	83	1	1	2	2	1	15	3	0	2	−	2	2	2	2	2
21	93	2	1	1	3	−	13	4	0	−	−	2	2	2	2	2
22	84	1	1	1	2	17	41	4	3	−	8	1	2	2	1	2
23	76	2	1	2	1	6	17	4	3	4	1	2	2	2	2	2
24	89	2	1	1	1	8	29	4	3	−	14	2	2	2	2	2
25	82	2	1	1	1	9	19	4	2	4	7	2	2	2	2	2
26	85	2	1	1	2	16	36	4	4	3	14	1	2	2	2	2
27	72	1	1	1	1	8	27	5	4	4	4	2	2	2	2	2
28	86	2	1	1	1	4	19	4	1	4	5	2	2	2	1	2
29	82	2	1	2	3		38	4	0	−	−	2	2	2	2	2
30	64	2	1	2	1	8	26	5	4	3	2	2	1	2	2	2
31	93	2	1	2	1	13	27	4	0	4	12	2	1	1	1	2
32	63	2	1	1	1	8	28	5	4	−	2	2	2	2	2	2
33	42	1	1	2	2	0	35	5	0	4	−	2	2	2	2	2
34	86	1	1	2	1	18	39	4	3	−	6	2	1	1	2	2
35	89	2	1	2	1	13	25	4	3	−		2	2	2	2	1
36	81	2	1	2	1	12	18	4	1	3	11	2	2	2	1	2
37	96	2	1	2	2	5	27	2	2	4	17	2	2	2	2	2
38	88	2	1	2	1	5	13	3	3	4	7	2	2	2	2	2
39	85	2	1	1	1	6	21	4	4	4	1	2	2	2	2	2
40	72	2	1	2	1	29	47	5	3	5	7	2	2	1	1	2
41	80	2	1	1	2	7	19	5	0	−	9	2	2	2	2	2
42	81	2	1	2	1	9	16	4	0	1	−	2	2	2	1	2
43	74	2	1	1	1	2	21	5	2	4	13	2	2	2	2	2
44	80	2	1	2	1	14	24	4	3	4	−	1	2	1	2	2
45	53	1	1	1	2	6	26	5	0	5	−	2	2	2	2	2
46	66	2	1	1	3	−	10	4	0	−	−	2	2	2	2	2
47	84	1	1	1	1	7	17	5	3	4	3	2	2	2	2	2
48	93	2	1	1	1	13	26	4	2	1	5	2	2	2	2	2
49	86	2	1	1	1	17	36	4	0	2	10	2	2	2	2	2
50	93	2	1	2	1	5	21	4	2	−	9	2	2	2	2	2
51	91	2	1	2	3	−	13	2	0	−	−	1	2	2	2	2
52	70	1	1	2	1	7	17	5	3	5	2	2	2	2	2	2
53	88	2	1	2	3	−	14	4	0	4	−	2	2	2	2	2
54	84	2	1	1	1	13	21	4	3	3	2	2	2	2	1	1
55	66	2	1	2	1	13	79	5	4	5	5	2	2	2	2	2
56	89	2	1	2	1	13	27	4	3	4	6	1	2	2	2	2
57	76	2	1	1	1	12	20	4	3	4	3	2	2	2	1	2
58	85	2	1	2	1	11	21	4	2	−	9	2	2	2	2	2
59	85	2	1	1	1	13	23	4	4	4	4	2	2	2	2	2
60	73	2	1	2	1	11	32	5	4	5	4	2	2	2	1	2
61	86	2	1	2	1	3	11	4	3	−	3	2	2	2	2	2
62	73	2	1	1	1	12	20	5	4	5	2	2	2	2	2	2
63	66	2	1	1	1	4	26	4	3	4	1	2	2	2	2	2
64	78	2	1	2	2	5	13	4	0	−	−	2	2	2	2	2
65	89	2	1	1	1	9	25	4	0	−	−	2	2	2	2	2
66	84	2	1	1	4	−		4	0	−	−	2	2	2	2	2
67	86	2	1	2	2	10	14	3	3	3	2	2	2	2	2	2
68	79	2	1	2	1	10	25	5	3	4	6	2	2	2	2	2
69	94	1	1	2	1	38	44	2	0	−	−	2	2	1	2	2
70	80	1	1	2	3	−	32	0	0	−	−	1	2	2	2	2
71	88	1	1	1	2	1	15	5	3	5	11	2	2	2	2	2
72	58	2	1	1	1	10	25	5	3	5	2	2	2	2	2	2
73	91	2	1	1	1	3	8	4	4	4	1	2	2	2	2	2
74	87	2	1	1	1	9	19	4	0	1	0	2	2	2	2	2
75	88	2	1	2	1	8	13	4	0	4	0	2	2	2	2	2
76	89	2	1	2	1	15	23	4	0	0	0	2	2	2	2	2
77	80	2	1	2	2	0	15	5	4	4	4	2	2	2	2	2
78	93	2	1	2	1	13	16	4	0	−	2	2	2	2	2	2
79	79	2	1	2	1	3	11	3	0	−	0	1	2	2	2	1
80	84	2	1	1	1	6	20	4	3	4	1	2	2	2	2	2
81	90	2	1	2	2	10	16	3	0	−	−	2	2	2	2	2
82	91	2	1	1	3	−	19	3	0	−	−	2	2	2	2	2
83	81	1	1	2	2	6	27	4	0	4		2	2	2	2	2
84	89	2	1	1	3	−	23	4	0	−		2	2	2	2	2
85	83	2	1	1	1	20	33	4	0	−	−	2	2	1	2	2
86	91	1	1	1	1	10	114	4	0	−1	−	2	2	2	2	2
87	87	1	1	2	2	9	17	4	3	4	5	2	2	2	2	2
88	69	2	1	2	1	3	10	5	4	5	4	2	2	2	2	1
89	82	2	1	1	1	33	56	4	3	3	13	2	2	2	1	1
90	82	2	1	2	1	14	25	3	3	−	8	2	2	2	1	1
91	87	2	1	1	3	−	10	3	0	−1	0	2	2	2	2	2
92	92	1	1	2	3	−	27	4	0	−	−	1	1	2	2	2
93	86	2	1	2	1	12	15	4	0	−	−	2	2	1	2	2
94	61	1	1	2	1	7	26	5	4	5	3	2	2	2	2	2
95	50	1	1	2	2	6	12	5	0	5	0	2	2	2	2	2
96	80	2	1	1	1	7	26	4	0	−	5	2	2	2	2	2
97	83	1	1	2	1	1	9	4	0	−	0	2	2	2	2	2
98	76	2	1	1	1	4	15	4	3	4	7	2	2	2	2	2
99	70	2	1	2	1	9	20	4	3	−	11	2	2	2	2	2
##	71	2	1	2	1	4	16	5	4	5	3	2	2	2	2	2
##	87	2	1	2	1	6	22	0	0	−		2	2	2	2	2
##	89	2	2	2	2	5	15	4	0	0	6	2	2	2	2	2
##	88	2	2	1	2	14	39	4	3	−	11	1	1	2	2	2
##	85	2	2	2	2	3	28	4	0	−1	−	2	2	2	2	2
##	88	2	2	2	2	1	16	4	0	4	−	2	2	2	2	2
##	85	2	2	1	2	5	21	4	3	3	5	2	2	2	2	1

##	89	2 2	2	2	3	17 -		0 -	-		2	2	2	2	1
##	90	1 2	1	2	3	11 -	-		-1 -		2	2	2	2	2
##	84	1 2	2	2	3	27	4	0 -	-		2	2	2	2	2
##	80	1 2	1	3 -		24	0	0	-1		1	2	2	2	2
##	89	2 2	2	2	0	30	4	3	3	20	2	2	1	2	2
##	81	2 2	1	3 -		5 -	-		-1 -		2	2	2	2	2
##	93	2 2	1	2	11	15	4 -	-	-		2	1	1	2	2
##	82	2 2	1	2	3	16	4	0 -	-		1	2	2	2	2
##	90	2 2	2	3 -		13	2	0 -	-		2	2	2	2	2
##	84	2 2	1	2	4	24	0	2 -		13	2	2	2	2	2
##	85	2 2	2	2	11	14	4	0	4 -		2	2	2	2	1
##	72	2 2	2	2	4	12	5	3	4	6	2	2	2	2	2
##	87	2 2	1	2	9	12	4	0 -	-		2	1	2	2	1
##	75	1 2	1	2	10	40	5	0 -	-		1	2	2	2	2
##	69	1 2	1	2	7	26	5	0	4 -		2	2	2	2	2
##	93	2 2	1	2	22	26	3	0 -	-		2	2	1	2	2
##	92	2 2	1	2	10	34	3	0 -	-		2	2	1	2	2
##	90	2 2	2	3 -		8	4	0 -	-		2	2	2	2	2
##	87	2 2	1	2	10	16	3	0	0	0	2	2	2	1	2
##	77	1 2	1	2	4	27	4	3	-1	18	2	2	2	2	2
##	64	2 2	1	2	6	29	5	0 -	-		2	2	2	2	2
##	88	2 2	1	2	6	15 -		0	-		2	2	2	2	1
##	87	2 2	2	3 -		10 -		0 -	-		2	2	2	2	2
##	90	2 2	1	2	7	17	3	0	0 -		1	2	2	2	2
##	86	1 2	1	2	3	6	5	0	4 -		2	2	2	2	2
##	84	2 2	1	2	6	28	3	0	1 -		2	2	2	2	2
##	69	1 2	1	2	2	26	5	0	4 -		2	2	2	2	2
##	86	2 2	1	2	3	12	4	0	2 -		2	2	2	2	2
##	86	2 2	1	2	8	17 -		0 -	-		2	1	1	2	2
##	94	2 2	2	2	2	15	3	0 -	-		1	2	2	2	2
##	45	1 2	1	2	2	16	5	0	4 -		2	2	2	2	2
##	95	2 2	2	2	2	13	4	0 -	-		2	2	2	2	2
##	95	2 2	1	2	3	15	4	0 -	-		2	2	2	2	2
##	84	2 2	2	2	7	20	4	3 -		2	2	2	2	1	1
##	75	2 2	2	2	10	28	5	0 -			2	2	2	2	1
##	84	2 2	2	2	2	10	5	3	4	11	2	2	2	2	2
##	69	2 2	1	2	3	17	5	4	5	2	2	2	2	2	2
##	84	2 2	2	2	3	12	4	3 -		5	2	2	2	2	2
##	78	2 2	2	2	5	18	4	0 -	-		2	2	2	2	2
##	32	2 2	1	2	24	31	5	4	4	13	2	2	1	2	2
##	96	1 2	2	2	6	9	4	0 -	-		2	2	2	2	2
##	85	2 2	1	2	17	27	4	0 -	-		2	1	2	1	2
##	89	1 2	2	2	20	34	4	0	4 -		2	2	2	2	2
##	57	1 2	2	2	1	16	5	3	5	3	2	2	2	2	2
##	89	2 2	1	2	0	19	4	3	4	16	2	2	2	2	2
##	84	1 2	2	2	13	23	4	0 -	-		2	2	2	1	2
##	93	2 2	1	2	6	11	4	0 -			2	2	2	2	2
##	72	2 2	2	2	3	17	5	0	4 -		2	2	2	2	2
##	90	2 2	1	3 -		17	3	0 -			2	2	2	2	2
##	95	2 2	2	2	3	12	4	0 -			2	2	2	2	2
##	89	2 2	1	2	6	28	3	0 -			2	2	2	2	2
##	89	2 2	2	3 -		6	1	-			2	1	2	2	2
##	82	2 2	2	2	0	18	4	0	4	5	2	2	2	2	2
##	93	1 2	1	2	6	29	4	0 -			2	2	2	2	2
##	83	2 2	1	2	1	12	4	0 -			2	2	2	2	2
##	84	2 2	2	2	1	13	4	3	4	2	2	2	2	2	2
##	90	1 2	1	3 -		14	4	-			2	2	2	2	2
##	86	2 2	1	2	4	12	4	2 -			2	2	2	2	2
##	80	1 2	1	2	2	17	4	0	3 -		2	2	2	2	2
##	90	2 2	2	3 -		12	3	-			1	2	2	2	2
##	81	1 2	1	2	16	24	4	0	4 -		1	1	1	2	2
##	87	2 2	1	2	0	18	4	0 -			2	2	2	2	2
##	68	1 2	1	2	15	69	5	4	5	28	2	2	1	2	2
##	90	1 2	2	2	3	10	4	0 -			2	2	2	2	2
##	90	2 2	1	2	0	14	4	0	4 -		2	2	2	2	2
##	71	2 2	1	2	15	36	5	0 -			2	2	1	2	1
##	98	2 2	1	2	0	11	4	0 -			2	2	2	2	2
##	67	2 2	2	2	0	10	5	0 -			2	2	2	2	2
##	99	2 2	1	2	2	10	4	0	4 -		2	2	2	2	2
##	76	1 2	2	2	4	16	5	4	5	4	2	2	2	2	2
##	87	2 2	2	3 -		4	4	0	-1 -		2	2	2	2	2
##	90	2 2	2	2	11	20	4	0			2	2	2	1	2
##	82	2 2	1	2	7	13	4	0 -	-		2	2	2	1	2
##	62	1 2	1	1	18	35	5	3	4	10	1	2	2	1	2
##	84	1 2	2	2	3	10	4	0 -	-		2	2	2	2	2
##	86	2 2	2	2	10	18	3	3	3	2	2	2	2	2	2
##	97	2 2	2	2	0	12	1	0 -	-		2	2	2	2	2
##	81	2 2	1	2	6	14	5	0 -		2	2	2	2	2	2
##	93	1 2	2	2	16	29	4	0	3 -		1	2	2	2	2
##	82	2 2	2	2	9	16	1	0 -	-		2	1	2	2	2
##	52	2 2	1	2	9	9	5	0	5 -		2	2	2	2	2
##	86	2 2	1	2	10	20	5	0	4 -		2	2	2	2	2
##	92	2 2	1	2	9	16	4	3	3 -		2	2	1	2	2
##	79	1 2	2	2	12	26	4	3 -		7	2	2	2	2	1
##	90	2 2	2	2	14	25	3	0 -	-		2	2	1	2	2
##	91	2 2	1	2	0	42	4	3	4	11	2	2	2	2	2
##	84	1 2	2	3 -		8	4	0 -		0	2	2	2	1	2
##	76	1 2	1	2	0	7	3	0 -		0	2	2	2	2	2
##	88	1 2	1	2	8	16	4	0	0	0	2	2	2	2	2
##	87	2 2	1	2	1	20	3	0 -			2	2	2	2	2
##	92	2 2	2	2	0	9	3	0 -		16	2	2	2	2	2
##	78	2 2	2	2	9	18	4	0	4		2	2	2	1	2
##	89	1 2	1	2	7	17	5	3	-1	6	2	2	2	2	2
##	94	2 2	1	2	13	22	4	0	4		2	2	2	1	2
##	96	2 2	2	2	3	27	3	0 -			2	2	2	2	2
##	81	2 2	1	3 -		3	0	0 -			2	2	2	2	2
##	92	2 2	1	2	3	17	4	0	0 -		2	2	2	2	2
##	86	2 2	2	2	8	16	4	0	3 -		2	2	2	2	2
##	88	2 2	2	3 -		21	4	0 -			2	2	2	2	2
##	90	2 2	2	2	9	16	4	0	4 -		2	2	2	2	2
##	92	2 2	1	2	0	20	4	3 -		6	2	2	2	2	2
##	90	2 2	2	2	11	22	4	3 -			2	2	2	2	2
##	73	2 2	2	2	1	16	5	0	5 -		2	2	2	2	2
##	73	2 2	2	2	31	46	4	0	0 -		2	2	2	1	1
##	90	1 2	2	3 -		9	5	0 -	-		2	2	2	2	2
##	87	2 2	2	2	2	13	1	0	1 -		2	2	2	2	2
##	88	2 2	2	3 -		4	0	0	0 -		2	2	2	2	2
##	88	1 2	2	3 -		24	4	0	3		1	2	1	2	1
##	77	2 2	2	2	0	16	4	4	4	1	2	2	2	2	2

##	96	2	2	1	2		22	12	3	0	-		-		2	2	2	2	2
##	87	1	2	2	2		10	17	3	2		3		5	2	2	2	2	2
##	68	1	2	2	3	-		15	3	0		0	-		2	2	2	2	2
##	76	1	2	1	2		0	8	3	0		0	-		2	2	1	2	2
##	85	2	2	1	2		13	28	4	0	-		-		2	1	2	2	2
##	84	2	2	1	2		10	25	4	3	-			3	2	2	2	1	2
##	69	2	2	1	2		0	11	5	0		5	-		2	2	2	2	2
##	91	2	2	1	3	-		10	3	0	-		-		2	2	2	2	2
##	90	2	2	2	3	-		2	4	0	-		-		2	2	2	2	2
##	43	2	2	1	1		26	32	5	0		5	-		2	2	1	2	2
##	71	2	2	2	2		8	19	4	0		4	-		2	2	2	2	2
##	48	1	2	1	2		0	10	5	0		4	-		2	2	2	2	2
##	85	2	2	2	2		0	7	4	0	-		-		2	2	2	2	2
