# Supplementary material for: Functional outcomes after the treatment of hip fracture
Source: PLoS One. 2020 Jul 30;15(7):e0236652. doi: 10.1371/journal.pone.0236652 (PMC7392284; doi:10.1371/journal.pone.0236652)
Supplement: Attachment — Submitted filename: Point-by-Point Responses2.docx [file pone.0236652.s003.docx]

Point-by-Point Responses to Comments of Reviewer #1

We would like to thank Reviewer #1 for evaluating our manuscript. We added discussions about mortality, length of presurgical duration, and factors associating with walking ability, resulting in changes from Discission lines 150 to 161, 178 to 185, and lines 167 to 178. Also, the description of rehabilitation was changed by citing some recent randomized controlled trials (lines 186 to 189 and 196 to 201).

Point-by-Point Responses to Comments of Reviewer #2

We thank Reviewer #2 for evaluating our manuscript. We have revised the content according to the advice provided. Our responses to the reviewer’s comments are provided below. The reviewer’s comments are indicated by underlined text.

1. Point 6 in methodology, “ retrospective medical record evaluation” this is not a study design, the study design of this research is “ retrospective cohort study”.

We have revised the text accordingly (line 63 to 65).

Discussion:

2. Presentaion of the discussion should be done with the study presentation first then to compare with other studies, not the reverse.

We have revised the text accordingly.

3. There is no discussion for morbidity or mortality factors

We added the discussion about mortality rate in lines 150 to 161.

4. No discussion with the hospital stay,……

We added the discussion about presurgical duration in lines 178 to 185. In this study, there was no correlation wit hospital stay and functional recovery, so we are not discussing length of hospital stay.

5. No discussion with factors related to the walking ability.

We added the discussion about factors related to the walking ability in lines 167 to 178.

6. In pages 17& 18, lines 240 to 262: are not related to presented data. In this study there are no evaluation of the rehabilitation done for patients in both groups. Could be written in two to three lines only. Write recommendation related to results.

We omitted the item on rehabilitation content and revised it by citing some recent randomized controlled trials. (lines 186 to 189 and 196 to 201).
